# Handwriting training in Parkinson’s disease: A trade-off between size, speed and fluency

**DOI:** 10.1371/journal.pone.0190223

**Published:** 2017-12-22

**Authors:** Evelien Nackaerts, Sanne Broeder, Marcelo P. Pereira, Stephan P. Swinnen, Wim Vandenberghe, Alice Nieuwboer, Elke Heremans

**Affiliations:** 1 Department of Rehabilitation Sciences, KU Leuven, Leuven, Belgium; 2 Department of Physical Education, São Paulo State University, São Paulo, Brazil; 3 Department of Movement Sciences, KU Leuven, Leuven, Belgium; 4 Department of Neurosciences, KU Leuven, Leuven, Belgium; 5 Department of Neurology, University Hospitals Leuven, Leuven, Belgium; The University of Melbourne, AUSTRALIA

## Abstract

**Background:**

In previous work, we found that intensive amplitude training successfully improved micrographia in Parkinson’s disease (PD). Handwriting abnormalities in PD also express themselves in stroke duration and writing fluency. It is currently unknown whether training changes these dysgraphic features.

**Objective:**

To determine the differential effects of amplitude training on various hallmarks of handwriting abnormalities in PD.

**Methods:**

We randomized 38 right-handed subjects in early to mid-stage of PD into an experimental group (n = 18), receiving training focused at improving writing size during 30 minutes/day, five days/week for six weeks, and a placebo group (n = 20), receiving stretch and relaxation exercises at equal intensity. Writing skills were assessed using a touch-sensitive tablet pre- and post-training, and after a six-week retention period. Tests encompassed a transfer task, evaluating trained and untrained sequences, and an automatization task, comparing single- and dual-task handwriting. Outcome parameters were stroke duration (s), writing velocity (cm/s) and normalized jerk (i.e. fluency).

**Results:**

In contrast to the reported positive effects of training on writing size, the current results showed increases in stroke duration and normalized jerk after amplitude training, which were absent in the placebo group. These increases remained after the six-week retention period. In contrast, velocity remained unchanged throughout the study.

**Conclusion:**

While intensive amplitude training is beneficial to improve writing size in PD, it comes at a cost as fluency and stroke duration deteriorated after training. The findings imply that PD patients can redistribute movement priorities after training within a compromised motor system.

## Introduction

Parkinson’s disease (PD) is a neurodegenerative disorder characterized by the loss of dopaminergic neurons in the basal ganglia leading to a number of motor symptoms [1]. Aside from the primary motor symptoms, handwriting difficulties occur frequently and are generally known as micrographia, i.e. a reduction in writing amplitude [2]. These difficulties commonly result in reduced legibility. While micrographia is one of the important features of handwriting impairment in PD, it is not the only deficit [3]. Letanneux *et al*. showed that three other variables may also differ from healthy controls, i.e. stroke duration, velocity and fluency [3]. Altogether, smaller writing size, longer stroke duration, slower handwriting velocity and reduced fluency point to dysgraphic handwriting [4–9], which characterizes performance in PD better than micrographia alone. Treatment with dopaminergic medication or deep brain stimulation was found to have a beneficial effect on the latter three variables, but not on amplitude [10–13]. As such, there is an unmet need for rehabilitation interventions that target writing size, which can be considered as the most important parameter determining handwriting legibility. In a recent randomized controlled trial, our group showed solid improvements of writing amplitude after six weeks of intensive amplitude training, in addition to dopaminergic medication [14].

In healthy persons, amplitude and speed during voluntary arm and hand movements, such as writing, obey certain generally accepted rules of motor control: irrespective of the muscles involved, larger movement amplitudes are accompanied by an increase in velocity [15]. However, in patients with PD this speed-amplitude relation was shown to be altered and characterized by either a reduced movement speed to maintain amplitude or by dysregulation of movement size to the benefit of movement speed in comparison to healthy controls [7, 16–18], expressing the symptomatic effects of bradykinesia. Also, patients with PD modulated acceleration measures inefficiently as compared with controls, i.e. had a lower mean acceleration and smaller peak acceleration compared to controls, mainly when they were requested to write with large stroke sizes [7]. Early EMG studies provided a partial explanation for this difference. While healthy persons needed a single EMG burst to execute arm movements, patients with PD required multiple bursts, a pattern which was aggravated during large-amplitude movements [19–22].

Previous studies in PD using graphics tablets have also reported prominent problems in movement fluency (see Letanneux *et al*. for a review [3]). Writing fluency can be measured by the normalized jerk of a writing trajectory, which reflects the change in acceleration normalized for different stroke durations and sizes [6]. An increased normalized jerk during several handwriting tasks was found consistently in PD, reflecting a reduced capacity to coordinate the fingers and wrist [6]. Most studies in this field investigated the speed, amplitude and fluency of writing during single session experiments, whereby writing at different sizes, velocities and complexity was compared [3]. So far, it has not been investigated how training of handwriting, with a focus on improving writing amplitude, may differentially affect writing size, stroke duration, speed and fluency and the relation among these variables after a multi-session training. Gaining insight into the effects of training on these parameters is crucial to refine future rehabilitation interventions for handwriting problems in PD.

## Methods

### Participants and study design

The same patient groups were included as described in an earlier paper by Nackaerts *et al*. [14], of which this study comprises a more detailed kinematic analysis. In short, 38 right-handed patients with PD participated [23]. Inclusion criteria were: (i) diagnosis of PD according to the United Kingdom PD Society Brain Bank criteria [24]; (ii) Hoehn and Yahr (H&Y) stage I to III in the on-phase of the medication cycle [25]; (iii) experiencing writing problems, as identified by a score of one or more on item 2.7 (Handwriting) of the Movement Disorder Society-sponsored revision of the Unified Parkinson’s Disease Rating Scale (MDS-UPDRS) part II [26]; and (iv) the absence of severe cognitive impairment (Mini-Mental State Examination (MMSE) ≥ 24) [27]. Exclusion criteria were: (i) color blindness or other impairments in vision interfering with handwriting; (ii) upper limb problems unrelated to PD; and (iii) deep brain stimulation.

Patients were assigned to one of two training programs by means of a stratified randomization procedure based on H&Y stage and age. Eighteen patients were assigned to an intensive writing amplitude training (= EXP group) and 20 to a placebo group, who received a stretch and relaxation program designed not to influence writing amplitude (= PLB group). Both programs were equally time-intensive and included 30 minutes of practice, five days per week over the course of six weeks. All patients filled out a diary so that the amount and duration of the intervention could be closely monitored. As well, patients in both groups received a weekly follow-up by the researcher, during which feedback and support were provided. In short, writing training consisted of pen-and-paper exercises and exercises on the touch-sensitive tablet and was aimed at improving writing amplitude with the help of visual cues. The stretch and relaxation program aimed to teach patients how to alleviate tension in the upper limbs. More detailed information on general characteristics of both groups and the content of the training programs can be found in Nackaerts *et al*. [14].

The study design and protocol were approved by the local Ethics Committee of the KU Leuven and were in accordance with the code of Ethics of the World Medical Association (Declaration of Helsinki, 1967). After explanation of the study protocol written informed consent was obtained from all participants prior to participation in the study. The trial was registered as ClinicalTrials.gov Protocol Record G.0906.11.

### Outcome measures

The effect of training on stroke duration (s), writing velocity (cm/s) and normalized jerk was measured on a touch-sensitive tablet (sampling frequency 200 Hz, spatial resolution 32.5 μm) before and after the training program and after a six-week retention period. During all tests, two types of tasks were performed, i.e. an automatization and transfer task [14]. In short, for the automatization task patients were asked to write a 3-loop sequence at two sizes, 0.6 or 1.0 cm indicated by visual target zones, and this either alone or while counting high or low tones [28]. The transfer task incorporated writing of a trained and untrained sequence in the presence and absence of the visual target lines, resulting in four conditions. The trained sequence consisted of the same 3-loop sequence mentioned above. The untrained sequence involved a continuous figure 8-like movement [14]. Both trained and untrained sequences were performed with and without cues and at the two sizes described above.

For both the automatization and transfer task, three blocks were performed in which each condition was provided in a random order. Each writing condition lasted 27 s and was followed by a rest period of 6 s. Participants were tested during the on-phase of the medication cycle at baseline, after six weeks of training (post-test) and after six weeks without training (retention-test). Medication intake was monitored and kept constant throughout the study.

### Data processing and statistical analysis

All data from the tablet were filtered at 7 Hz with a 4^th^-order Butterworth filter and further processed using Matlab R2011b. Stroke duration (s), writing velocity (cm/s) and normalized jerk were extracted as dependent variables. The size and duration of individual up- and downstrokes were defined by calculating local minima and maxima. Writing velocity (cm/s) was calculated based on the duration and size of the individual strokes. The normalized jerk was calculated as the change in acceleration, normalized for different stroke durations and sizes making in a unit-free measure, using the formula described by Teulings *et al*. [6]: $Normalized\;jerk=\sqrt{\frac{1}{2}\int dt\;j^{2}(t)\times \frac{{duration}^{5}}{{length}^{2}}}$, where j(t) is the third time derivative of position.

Statistical analysis was performed using SPSS software (version 24). Both the automatization and transfer task were analyzed using a mixed model approach. For the automatization task Group (EXP or PLB), Time (baseline, post-training or retention) and Task (single or dual) were used as fixed factors and MAM-16 as a covariate, as it differed between both patient groups. To study transfer, Group, Time, Task (trained or untrained) and Cue (cued or uncued) were incorporated as fixed factors, with MAM-16 as a covariate. Both models controlled for the within-subject differences by including random effects for participants. Finally, partial correlation analyses, corrected for MAM-16, were performed between writing parameters at baseline and clinical characteristics, i.e. disease duration, cognition (MMSE) and disease severity (MDS-UPDRS total score and UL tremor and sequence subscores). Similarly, the difference between baseline and post-training and between baseline and retention for the writing parameters were correlated to the clinical characteristics for the EXP and PLB group separately. Significance levels for all tests were set at p < 0.05.

## Results

Both amplitude conditions showed comparable results. Therefore, only results from the large-amplitude condition are described in detail. For results on the small-amplitude condition we refer the reader to the **Supplementary results (S1 File)**, unless indicated otherwise.

### Automatization task

#### Duration

**Fig 1:**
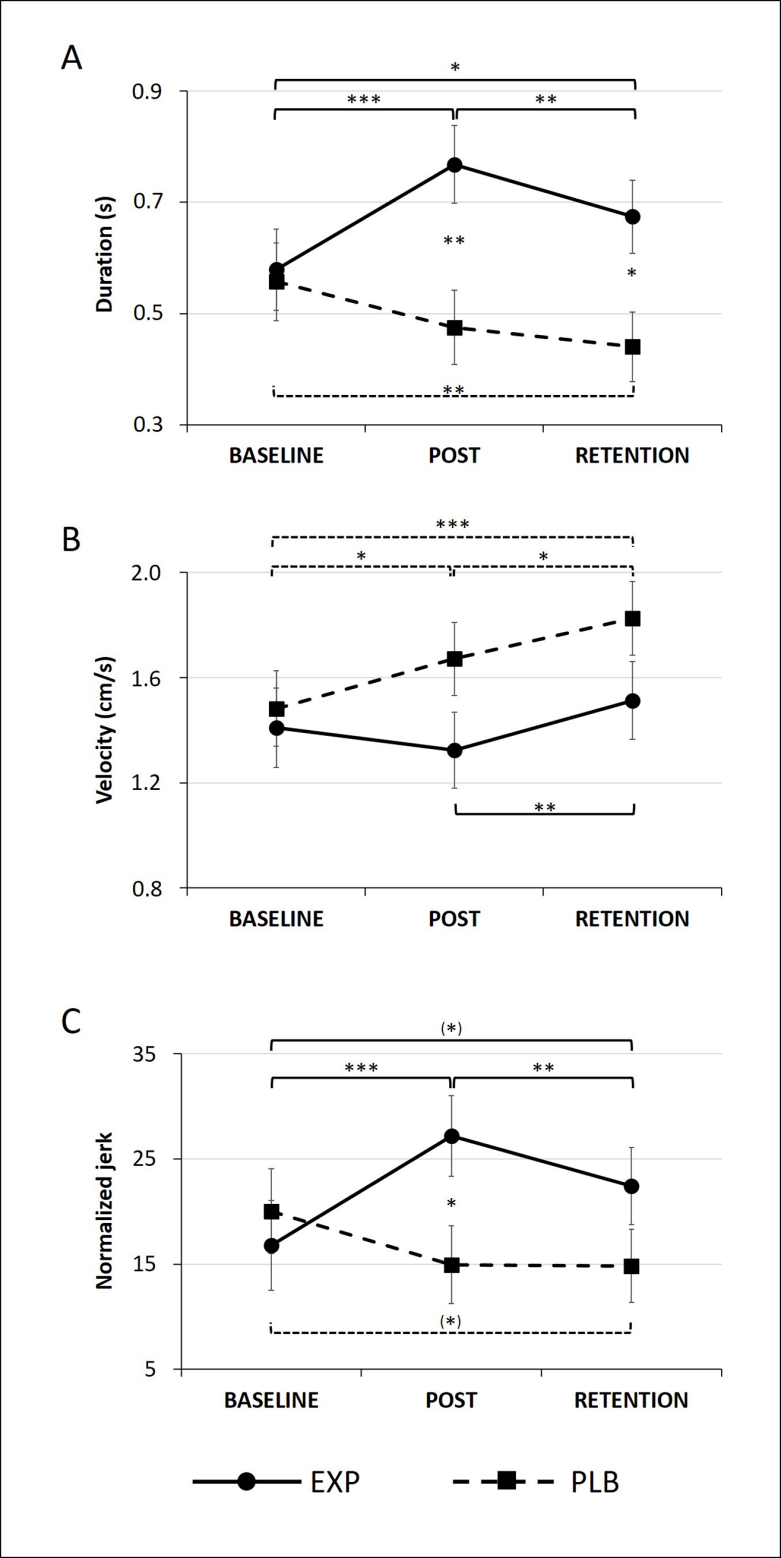
Automatization task in the large-amplitude condition. Mean and standard errors are displayed, corrected for MAM-16. (*) p < 0.1, * p < 0.05, ** p < 0.01, *** p < 0.001.

For writing duration, a significant Group x Time interaction was found in the large-amplitude condition (F = 11.434, p < 0.001) (**Fig 1A**). Post hoc analysis showed that the EXP group had a longer stroke duration compared to the PLB group post-training (p = 0.004) and at retention (p = 0.015). In the EXP group there was an increase in duration from baseline to post-training (p < 0.001) and retention (p = 0.023), although duration decreased again from post-training to retention (p = 0.004). A significant decrease in duration from baseline to retention (p = 0.003) was found in the PLB group.

#### Velocity

Also for velocity, the large-amplitude condition revealed a Time x Group interaction (F = 4.810, p = 0.011) (**Fig 1B**). Post hoc analysis showed no changes from baseline to post-training and an increase in velocity from post-training to retention in the EXP group (p = 0.002). On the other hand there was an increase in the PLB group from baseline to post-training (p = 0.011) and retention (p < 0.001) and from post-training to retention (p = 0.010).

#### Normalized jerk

A significant Time x Group interaction was found for the normalized jerk in the large-amplitude condition (F = 8.928, p < 0.001) (**Fig 1C**). Further analysis revealed a higher normalized jerk in the EXP group compared to PLB group post-training (p = 0.027). Additionally, the normalized jerk increased from baseline to post-training (p < 0.001) and retention (p = 0.054) in the EXP group, although there was a decrease again from post-training to retention (p = 0.005). In the PLB group on the other hand, the normalized jerk tended to decrease from baseline to retention (p = 0.069). A similar, though weaker pattern was found for the small amplitude (**S1 File**).

#### Correlation analysis

**Fig 2:**
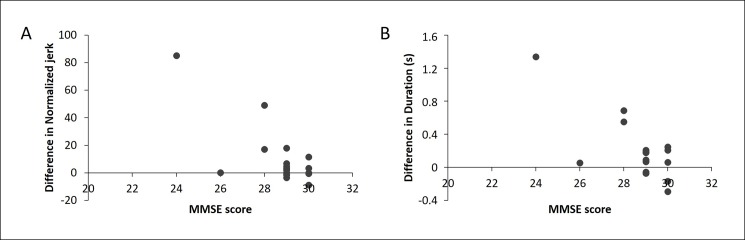
Correlation between the difference from baseline to post-training on the automatization task and cognition. (A) A greater difference in normalized jerk correlates with a lower MMSE score in the experimental group; (B) A greater difference in movement duration correlates with a lower MMSE score in the experimental group. Represented values are not corrected for MAM-16.

Correlation analysis revealed that, at baseline, a higher normalized jerk correlated with more difficulties on the upper limb sequence items of the MDS-UPDRS-III (r = 0.391, p = 0.017). When looking at the effects of training, it was found that a greater increase in the normalized jerk correlated with a lower MMSE score (post-training: r = -0.734, p = 0.001; retention: r = -0.676, p = 0.003) (**Fig 2A**) and with more difficulties on the upper limb sequence items of the MDS-UPDRS-III (post-training: r = 0.462, p = 0.062; retention: r = 0.579; p = 0.015) for the EXP group only. Additionally, in the EXP group a greater increase in duration was correlated to more cognitive difficulties (post-training: r = -0.732, p = 0.001; retention: r = -0.624, p = 0.007) (**Fig 2B**). No correlations were found between the outcomes and the MDS-UPDRS-III upper limb tremor items.

As the correlations with MMSE in the EXP group might have been driven by outliers, we performed a sensitivity analysis excluding the two outliers. This resulted in similar outcomes (**S1 File**).

### Transfer task

#### Duration

**Fig 3:**
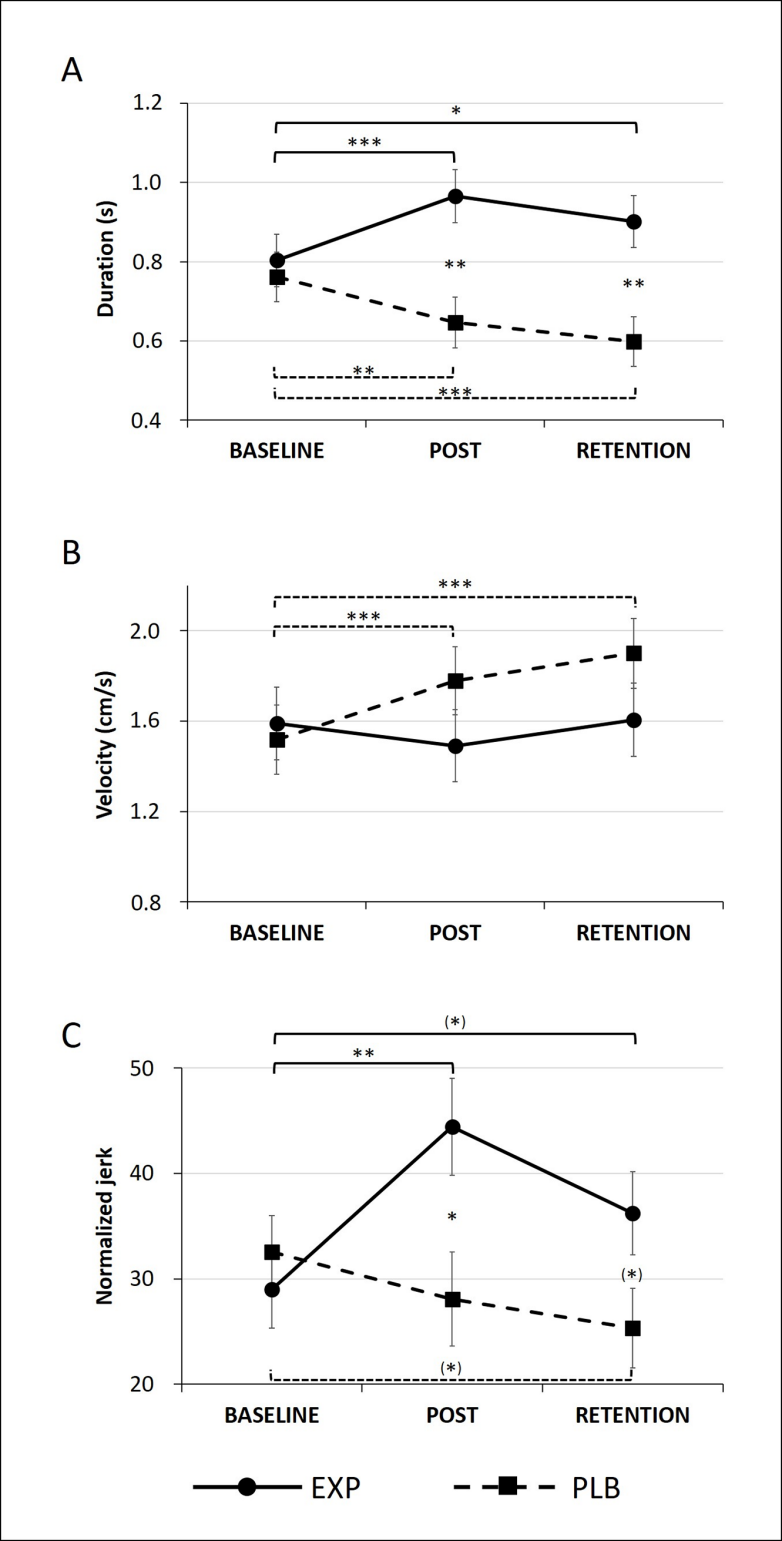
Transfer task in the large-amplitude condition. Mean and standard errors are displayed, corrected for MAM-16. (*) p < 0.1, * p < 0.05, ** p < 0.01, *** p < 0.001.

A significant Time x Group interaction was apparent in the large-amplitude condition (F = 18.237, p < 0.001) (**Fig 3A**), indicating a longer duration in the EXP compared to PLB group post-training (p = 0.001) and at retention (p = 0.002) at post hoc analysis. These differences were driven by an increase in duration in the EXP group from baseline to post-training (p < 0.001) and to retention (p = 0.023), in combination with a decrease in duration in the PLB group from baseline to post-training (p = 0.007) and to retention (p < 0.001).

#### Velocity

A significant Time x Group interaction was found for the large-amplitude condition (F = 13.888, p < 0.001), revealing a significant increase from baseline to post-training and retention in the PLB group (both p < 0.001) (**Fig 3B**). For the small-amplitude, post hoc analysis exposed an additional decrease in velocity from baseline to post-training in the EXP group (p = 0.036) (**S1 File**).

Finally, in the large-amplitude condition a Group x Cue interaction was found (F = 4.572, p = 0.033), indicating that patients in the PLB group wrote faster in the cued compared to uncued condition (p = 0.002).

#### Normalized jerk

In line with the automatization task, the transfer task also showed a significant Time x Group interaction for the large-amplitude condition (F = 8.732, p < 0.001) (**Fig 3C**). There was a higher normalized jerk in the EXP compared to PLB group post-training (p = 0.012) and at retention (p = 0.052). In addition, there was an increase in normalized jerk of the EXP group from baseline to post-training (p = 0.001). Also, there was a tendency to increase normalized jerk in the EXP group (p = 0.078) and decrease the normalized jerk in the PLB group (p = 0.066) from baseline to retention. A similar, though weaker pattern was found for the small amplitude (**S1 File**).

#### Correlation analysis

Correlation analysis showed that a higher normalized jerk was associated with more difficulties on the upper limb sequence items of the MDS-UPDRS-III (r = 0.346, p = 0.036), in line with results from the automatization task. Additionally, less fluent handwriting correlated with worse cognition (r = -0.324, p = 0.051). When looking at the effects of training, it was found that a greater increase in the normalized jerk correlated with a lower MMSE score (post-training: r = -0.550, p = 0.022) for the EXP group only. No correlations were found with the MDS-UPDRS-III upper limb tremor items.

As the correlations with MMSE in the EXP group might have been driven by outliers, we performed a sensitivity analysis excluding the two outliers. This resulted in similar outcomes (**S1 File**).

## Discussion

The present study revealed that, in addition to the previously detected improvement in writing size [14], other writing features are modified by intensive amplitude training. These adaptations included an increase in movement duration and a decrease in writing fluency across tasks and conditions, i.e. in both single and dual task, trained and untrained or cued and uncued sequences. On the contrary, the placebo group did not show beneficial effects on writing amplitude [14]. However, movement duration decreased, which led to an increase in writing velocity regardless of condition. Additionally, the placebo group showed an improvement of writing fluency in the automatization task.

The training of amplitude is a common rehabilitation approach in patients with PD, which was originally applied to improve speech deficits [29]. This treatment concept, known as Lee Silverman Voice Treatment (LSVT®), underlies the more recently developed protocol called ‘Training BIG’, to improve movement amplitude in the limbs [30, 31]. During BIG, amplitude is chosen as the main focus of training to overcome bradykinesia/hypokinesia by a ‘recalibration’ of the patients’ perception of normal amplitude execution. This amplitude focus is combined with an increase of intensity and complexity during training BIG, which is similar to the writing protocol used in this study. Interestingly, previous BIG trials showed positive effects on both the speed and amplitude during reaching movements, balance and bed mobility [30, 32, 33], except for one study on gait. In the latter, only stride length increased after training without increases in velocity or cadence in the as-fast-as-possible condition, confirming the possibility of a ceiling effect [30]. Importantly, none of the BIG trials analyzed the possible cost of amplitude-based training on motor fluency, cognitive load and energy consumption. Also, BIG training effects were largely studied on the trained tasks only, while in this study we analyzed trade-off and learning effects during automatization and transfer tasks. Finally, the differences between the training BIG studies and the current study could be a result of the task, as handwriting is a highly visually-driven movement incorporating both habitual and goal-directed motor control [34, 35].

**Fig 4:**
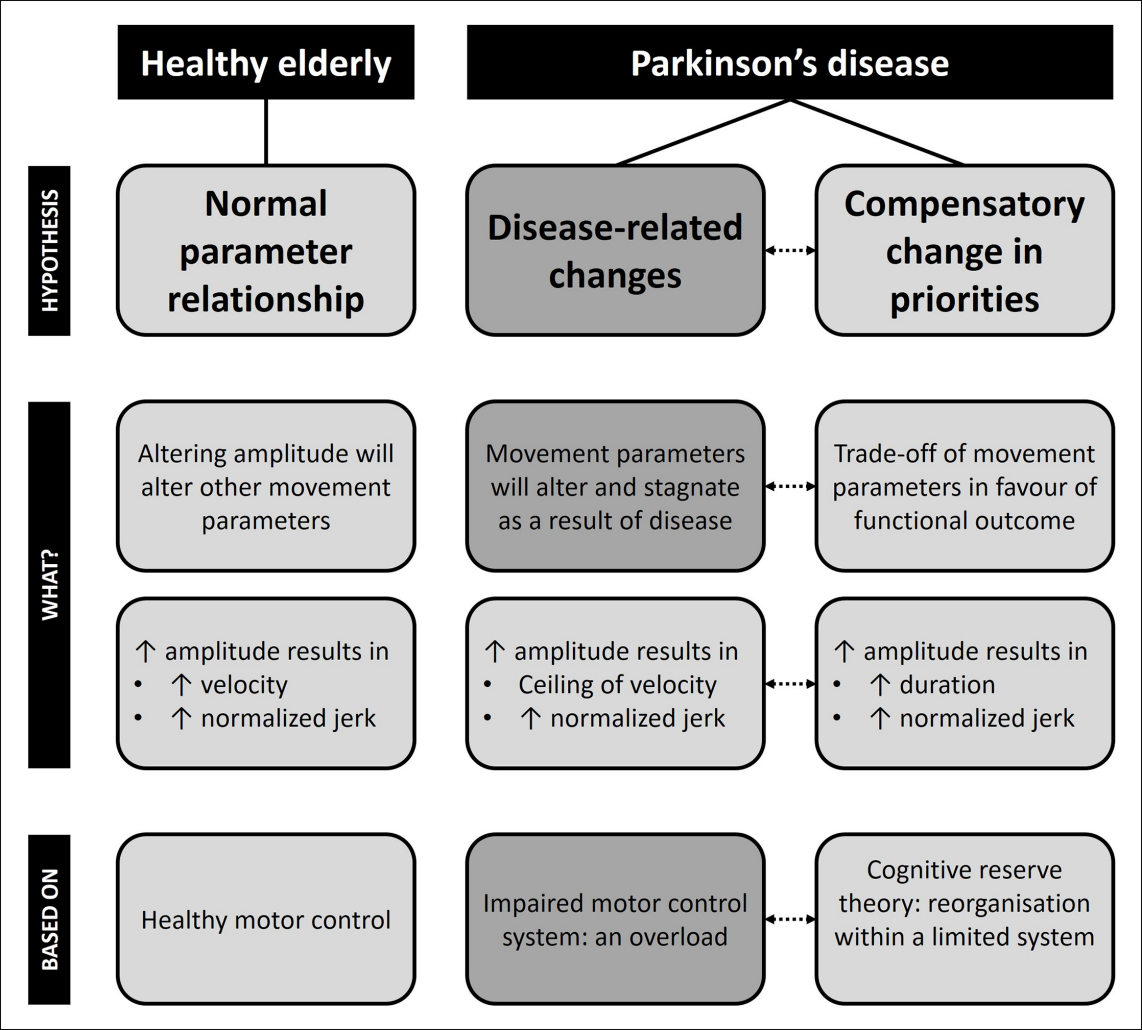
Movement parameter trade-off in health and disease. Dotted arrows indicate the possible interaction between disease-related changes and compensatory change in priorities.

We will interpret these results in the light of a proposed framework of movement parameter trade-off presented in **Fig 4**. Though not assessed in this study, the literature suggests that in a healthy system simply changing one movement parameter in one direction has an effect on the other parameters [15] (**Fig 4**, left panel). For instance, in healthy persons, movement speed was found to increase with rising amplitude [16, 36]. In contrast, the findings of the current and other studies on handwriting demonstrate that in patients with PD, velocity was found to saturate when amplitude increases (**Fig 4**, middle panel) [37–39]. The lack of concomitant increase in velocity (or velocity saturation) after amplitude training may be explained by a motor control impairment, affecting the speed-amplitude relation in patients with PD (**Fig 4**, middle panel). As a result, motor learning may come at a cost once patients reach the upper limits of their motor control system, explaining the stagnation in velocity. This view is supported by several studies showing that although patients with PD can alter one movement parameter, this occurs at the expense of another [4, 7, 40, 41].

A second explanation for the deterioration of movement duration is in line with the cognitive reserve theory, i.e. the recruitment of cognitive or other compensatory processes to substitute for ageing or pathology (for a review see Stern [42]) due to a priority change (**Fig 4**, right panel). This priority shift may also explain the increase of the normalized jerk accompanying the benefits on writing size, which may be a direct consequence of a system overload expressed as a loss of movement fluency. Worsening of writing fluency when movements are executed more slowly or with a larger amplitude is in agreement with previous studies in healthy young adults and other patient groups [43, 44]. Interestingly, in support of this hypothesis, we found that a worse cognitive profile and more advanced disease, represented by more sequential upper limb difficulties, resulted in a greater trade-off for movement duration and normalized jerk. This implies that rather than a ceiling, the lack of change in velocity may have been the result of the compensatory strategy used, which impacted on fluency and speed.

A final explanation, not illustrated in **Fig 4**, is that the placebo treatment consisting of stretching and relaxation was in fact beneficial, as the writing tasks were performed faster and with a decrease in the normalized jerk for the automatization task, although these changes were not necessarily beneficial for legibility of handwriting [45]. One specific explanation for the changes in velocity and fluency could be that the stretch and relaxation program influenced tremor positively. However, neither the correlation analysis with the upper limb tremor scores of the MDS-UPDRS-III, nor previous research on mindfulness training [46] supports this line of reasoning. Moreover, other rehabilitation studies showed that stretching and relaxation was not beneficial for motor performance in PD [47]. Therefore, we consider this explanation as unlikely.

### Implications for rehabilitation

The most comprehensive interpretation of our results suggests that intensive amplitude training resulted in a trade-off between amplitude and movement duration and fluency. This implies that the compensatory strategy learned during training may come at a cost, which has consequences for clinical practice. This knowledge does not argue against motor learning or rehabilitation. Rather, it acknowledges that in patients with PD it is of utmost importance to select one single parameter which is crucial for the largest functional gains. When aiming to improve the legibility of handwriting, the main parameter to focus on seems to be movement amplitude. Progression of training could then be aimed to follow logical steps if the clinical profile of the patients allows it. One strategy could be to solely target the most important movement parameter during the entire program, as is currently done in BIG programs [30]. This approach is based on the hypothesis that the parkinsonian brain may solve kinematic challenges in separate steps. Improvements will mainly be found on the parameter to which main priority is given, inevitably coming at a cost regarding the secondary parameters. However, as training progresses, resources may gradually become available again and the deterioration of other variables will decrease, optimizing the functional outcome of the training program. We also found that movement duration decreased, velocity increased and fluency improved again in the experimental group from post-training to retention for the automatization task, in combination with a stable amplitude [14]. It is, however, important to note that these effects were detected after a period without practice.

## Conclusion

In conclusion, we found that intensive amplitude training, reported earlier to help writing amplitude, came at the expense of writing duration and fluency in patients with PD. Several hypotheses were put forward to explain the observed trade-off. While there likely is an interaction between the disease-related and the compensatory changes, our findings point towards a reorganization within the limited motor control system due to changing motor priorities. Further research is indicated to investigate whether a more extensive training program could address various aspects of handwriting comprehensively. Until then, we advise to focus on amplitude to improve legibility of handwriting in PD.

## Supporting information

S1 File Supplementary results of the small-amplitude condition. (PDF)

S1 Dataset (XLSX)
