# Supplementary material for: Handwriting training in Parkinson’s disease: A trade-off between size, speed and fluency
Source: PLoS One. 2017 Dec 22;12(12):e0190223. doi: 10.1371/journal.pone.0190223 (PMC5741263; doi:10.1371/journal.pone.0190223)
Supplement: S1 File — (PDF) [file pone.0190223.s001.pdf]

Supplementary results: small-amplitude condition

Automatization task

Duration

For writing duration, a significant Group x Time interactions was found in the small- amplitude condition ($F = 12.341$, $p < 0.001$). Post hoc analysis showed that the EXP group had a longer stroke duration compared to the PLB group post-training ($p = 0.007$) and at retention ($p = 0.025$). In the EXP group there was an increase in duration from baseline to post-training ($p < 0.001$) and retention ($p = 0.053$), although from post-training to retention duration decreased again ($p = 0.002$). Additionally, the PLB group presented with a decrease in stroke duration from baseline to post-training ($p = 0.019$) and retention ($p = 0.001$).

Velocity

In the small-amplitude condition, a Time x Group interaction ($F = 5.088$, $p = 0.007$) was found for velocity. Similar to the large-amplitude condition, post hoc analysis exposed no changes from baseline to post-training and an increase in velocity from post-training to retention in the EXP group ($p = 0.001$). On the contrary, there was an increase in the PLB group from baseline to post-training ($p = 0.020$) and retention ($p < 0.001$) and from post-training to retention ($p = 0.017$).

Normalized jerk

For the small- amplitude, a significant Time x Group interaction was found ($F = 8.249$, $p = 0.001$). Post hoc tests showed a trend towards an increasing normalized jerk from baseline to post-training ($p = 0.066$), followed by a decrease from post-training to retention ($p = 0.042$) in the EXP group. Moreover, there was a decrease in normalized jerk from baseline to post-training ($p = 0.003$) and to retention ($p = 0.003$) for the PLB group.

Correlation analysis

Correlation analysis revealed that, at baseline, a higher normalized jerk correlated with more difficulties on the upper limb sequence items of the MDS-UPDRS-III ($r = 0.399$, $p = 0.014$). When looking at the effects of training, it was found that a greater increase in the normalized jerk correlated with a lower MMSE score (post-training: $r = -0.667$, $p = 0.003$; retention: $r = -0.582$, $p = 0.014$) and with more difficulties on the upper limb sequence items of the MDS-UPDRS-III (post-training: $r = 0.415$, $p = 0.097$; retention: $r = 0.489$; $p = 0.046$) for the EXP group only. Additionally, a greater increase in duration was correlated to more cognitive

difficulties in the EXP group (post-training: r = -0.671, p = 0.003; retention: r = -0.518, p = 0.033). No correlations were found this the MDS-UPDRS-III upper limb tremor items.

Transfer task

Duration

A significant Time x Group interaction was found (F = 18.036, p < 0.001). Post hoc analysis revealed a longer duration in the EXP compared to PLB group post-training (p = 0.004) and at retention (p = 0.006). These differences were driven by an increase in duration in the EXP group from baseline to post-training (p < 0.001) and to retention (p = 0.028), in combination with a decrease in duration in the PLB group from baseline to post-training (p = 0.004) and to retention (p < 0.001).

Velocity

A significant Time x Group interaction was found for the small-amplitude condition (F = 14.503, p < 0.001). Similar to the large-amplitude condition, there was an increase in the PLB group from baseline to post-training and retention (both p < 0.001). Additionally, post hoc analysis exposed a decrease in velocity from baseline to post-training in the EXP group (p = 0.036).

Normalized jerk

The transfer task also showed a significant Time x Group interaction for the small-amplitude condition (F = 6.521, p = 0.002). A tendency was observed towards a higher normalized jerk post-training (p = 0.081) in the EXP group compared to PLB group. In addition, there was an increase in normalized jerk of the EXP group from baseline to post-training (p = 0.011).

Correlation analysis

Correlation analysis showed that a higher normalized jerk was associated with more difficulties on the upper limb sequence items of the MDS-UPDRS-III (r = 0.347, p = 0.035), in line with results from the automatization task. Additionally, less fluent handwriting was correlated with worse cognitive skills (r = -0.330, p = 0.046). When looking at the effects of training, it was found that a greater increase in the normalized jerk correlated with a lower MMSE score (post-training: r = -0.526, p = 0.030) for the EXP group only. No correlations were found with the MDS-UPDRS-III upper limb tremor items.

Supplementary results: correlation analysis with MMSE, without outliers

Statistically, two outliers were detected for the MMSE score in the EXP group. As such, the correlation analysis was performed without both subjects to examine whether the effects remained present.

Automatization task

Small-amplitude condition

Similar to the initial analysis (including the outliers), it was found that a greater increase in the normalized jerk (post-training: $r = -0.646$, $p = 0.009$; retention: $r = -0.702$, $p = 0.004$) and a greater increase in duration (post-training: $r = -0.641$, $p = 0.010$; retention: $r = -0.760$, $p = 0.001$) were correlated with a lower MMSE score for the EXP group. Additionally, it was found that a greater increase in writing velocity correlated with a higher MMSE score in the EXP group at retention ($r = 0.795$, $p < 0.001$).

Large-amplitude condition

Similar to the initial analysis (including the outliers), it was found that a greater increase in the normalized jerk (post-training: $r = -0.606$, $p = 0.017$; retention: $r = -0.599$, $p = 0.018$) and a greater increase in duration (post-training: $r = -0.658$, $p = 0.008$; retention: $r = -0.794$, $p < 0.001$) were correlated with a lower MMSE score for the EXP group. Additionally, it was found that a greater increase in writing velocity correlated with a higher MMSE score in the EXP group at retention ($r = 0.828$, $p < 0.001$).

Transfer task

Small-amplitude condition

Similar to the initial analysis (including the outliers), it was found that a greater increase in the normalized jerk (post-training: $r = -0.712$, $p = 0.003$; retention: $r = -0.697$, $p = 0.004$) correlated with a lower MMSE score for the EXP group. Additionally, a greater increase in duration (post-training: $r = -0.712$, $p = 0.003$; retention: $r = -0.788$, $p < 0.001$) was correlated with a lower MMSE score and a greater increase in writing velocity correlated with a higher MMSE score in the EXP group at retention ($r = 0.569$, $p = 0.027$).

Large-amplitude condition

Similar to the initial analysis (including the outliers), it was found that a greater increase in the normalized jerk (post-training: r = -0.718, p = 0.003; retention: r = -0.718, p = 0.003) correlated with a lower MMSE score for the EXP group. Additionally, a greater increase in duration (post-training: r = -0.774, p = 0.001; retention: r = -0.774, p = 0.001) was correlated with a lower MMSE score and a greater increase in writing velocity correlated with a higher MMSE score in the EXP group at retention (r = 0.608, p = 0.016).
